# Breast and Thyroid Surgery in 2021 and Beyond

**DOI:** 10.3390/jcm11102894

**Published:** 2022-05-20

**Authors:** Fausto Fama’

**Affiliations:** Department of Human Pathology in Adulthood and Childhood “G. Barresi”, University Hospital “G. Martino” of Messina, 98123 Messina, Italy; ffama@unime.it; Tel.: +39-0902212644

Several studies in the literature report the association between breast and thyroid pathologies; however, the underlying causes (genetic, environmental, hormonal or immunological) have not yet been well explicated. In addition to ionizing radiation (an adjuvant treatment, which is indispensable for the treatment of breast malignancy), autoimmunity and functional alterations of the immune system are also predisposing factors. Therefore, the synergy between specialists and the establishment of integrated breast–thyroid follow-up programs remains the basis for an early diagnosis and the right management of these pathology associations.

In recent years, both breast and thyroid surgery have seen significant improvements.

**Breast surgery**

Since 1981, breast surgery has been oriented towards the concept of conservative breast surgery, aiming to treat the neoplastic pathology without resorting to traditional demolition techniques by limiting both the amount of breast parenchyma that is removed and the extension of the axillary dissection, in particular, introducing the concept of the sentinel lymph node [1,2].

Thus, women were inclined to accept these surgical procedures to reduce their psychological trauma and, in recent years, always in this direction, oncoplastic breast surgery, which, with the same oncological outcomes, also allows the improvement of cosmetic outcomes, avoiding asymmetries with respect to the contralateral breast or poor aesthetic results [3].

Recent attention has also been paid to ectopic breast cancers developed along the so-called milk line, an embryological line of cell migration that extends from the groin to the axilla [4,5]

**Thyroid surgery**

At the beginning of the 2000s, some authors published [6] the first reports concerning thyroid surgery performed with innovative surgical instruments (e.g., radiofrequency or ultrasound scalpel); these were used to reduce complication rates (i.e., postoperative bleeding, parathyroid lesions and nervous lesions) [7,8,9], the operating time and the in-hospital stay [10,11].

Subsequently, thyroid surgery focused on alternative access routes to the traditional open transcervical one, with the purpose of improving aesthetic outcomes, and, in 2001, the minimally invasive video-assisted thyroidectomy (MIVAT), combining the endoscopic technique with an anterior cervical approach, was proposed [12]. After Witzel’s pioneering attempt at sublingual remote access, which was later abandoned due to its high complication rate [13], the most widely used remote and scarless techniques currently are transoral endoscopic thyroidectomy (TOET, which uses the inferior labial vestibule as an access route) [14,15,16], the bilateral axilla-breast procedure (BABA, which involves bilateral axillary and areolar dissection) [17] and the retroauricular procedure [18]. These techniques benefit from the magnified–expanded and three-dimensional surgical view, which allows the noble anatomical structures to be better identified. More recently, robotic surgery associated with remote access procedures [19,20,21] has further expanded the patient inclusion criteria for both benign and malignant disease, and it is now also used for pediatric patients as well as adults [22].

This Special Issue, “Breast and Thyroid Surgery in 2021 and Beyond”, is dedicated to collecting high-quality scientific contributions concerning the advancements of breast and thyroid surgery and to gaining a better understanding of the underlying causes of the pathological associations between the breast and thyroid.

Forma A. et al. presented a retrospective study with the aim of evaluating the safety of oncoplastic breast surgery compared with non-oncoplastic conservative breast surgery, pointing out the attention on the histopathological and immunohistochemical features of breast cancer in relation to commonly applied surgical techniques. In the subgroup of patients who underwent oncoplastic breast surgery, a statistically significant relationship with the majority of tumoral histotypes was shown, except for the infiltrating lobular carcinoma, and also with regard to the expression of estrogen and/or progesterone receptors, but not for the HER2 receptor. The rate of reoperations was similar in the two groups, demonstrating oncoplastic breast surgery to be effective and safe.

Chong K.H. et al. presented an interesting study in which they investigated a group of patients undergoing thyroidectomy for autoimmune thyroiditis compared to a group undergoing treatment for goiter, using the predictive efficacy of preoperative serum levels of the macrophage migration inhibitory factor (MIF) to appraise the difficulty of the surgical procedure. They found that the MIF levels were significantly increased in the autoimmune thyroiditis patient group, and this was associated with greater surgical difficulty and a higher rate of postoperative complications. Furthermore, the elevated levels of MIF were positively correlated with higher blood loss and the prolongation of the mean operative time, which indicates the high surgical complexity.

Overall, I am confident that the issues raised in this Editorial will further stimulate research in this area in order to increasingly improve and optimize clinical practice.
